# Author Correction: Catastrophic slab loss in southwestern Pangea preserved in the mantle and igneous record

**DOI:** 10.1038/s41467-022-29842-z

**Published:** 2022-04-19

**Authors:** Guido M. Gianni, César R. Navarrete

**Affiliations:** 1 Consejo Nacional de Investigaciones Científicas y Técnicas (CONICET), Capital Federal, Argentina; 2 Laboratorio Patagónico de Petro-Tectónica, Universidad Nacional de la Patagonia “San Juan Bosco”, Dpto. de Geología, F.C.N, Comodoro Rivadavia, Chubut Argentina; 3 Instituto Geofísico Sismológico Ing. Fernando Volponi (IGSV), Universidad Nacional de San Juan, San Juan, Argentina

**Keywords:** Geochemistry, Geodynamics

Correction to: *Nature Communications* 10.1038/s41467-022-28290-z, published online 04 February 2022.

The original version of this Article contained an error in the numbering of the reference list, with references 132–134 being 154–156 and references 135–156 being 132-153. This has been corrected in the PDF and HTML versions of the Article.

