# Supplementary material for: Catastrophic slab loss in southwestern Pangea preserved in the mantle and igneous record
Source: Nat Commun. 2022 Feb 4;13:698. doi: 10.1038/s41467-022-28290-z (PMC8817029; doi:10.1038/s41467-022-28290-z)
Supplement: Supplementary file 1 — Supplementary information [file 41467_2022_28290_MOESM1_ESM.pdf]

Supplementary Materials for

Catastrophic slab loss in southwestern Pangea preserved in the mantle and igneous record

Guido. M. Gianni*, César R. Navarrete

*Corresponding author. Email: guidogianni22@gmail.com

This PDF file includes:

Supplementary Text

Supplementary Figs. S1 to S10

Table S1

Data file S1(Separate file)

Data file S2 (Separate file)

Supplementary Text

Additional geochemical analysis

We provide additional tectonic discrimination diagrams from Hildebrand *et al.*[60] and Whalen and Hildebrand[61] (Supplementary Fig. S5: Nb vs. Y; Ta vs. Yb; Gd/Yb vs. Sr/Y; La/Yb vs. Nb+Y and Supplementary Fig. S6: Gd/Yb vs. Nb/Y; Sm/Yb vs. Nb+Y; Nb/Y vs. Nb+Y; Gd/Yb vs. Ta+Yb; Sm/Yb vs. Ta+Yb; Nb/Y vs. Ta+Yb). We have used these diagrams to show that there is a predominance of slab break-off geochemical signatures in the Permian-Triassic igneous rocks from the Choiyoi Magmatic Province and dominating magmatic arc signatures in Upper Carboniferous-lowermost Permian and Upper Triassic-Jurassic igneous rocks (Supplementary Figs. S5, S6, S8,and S9).

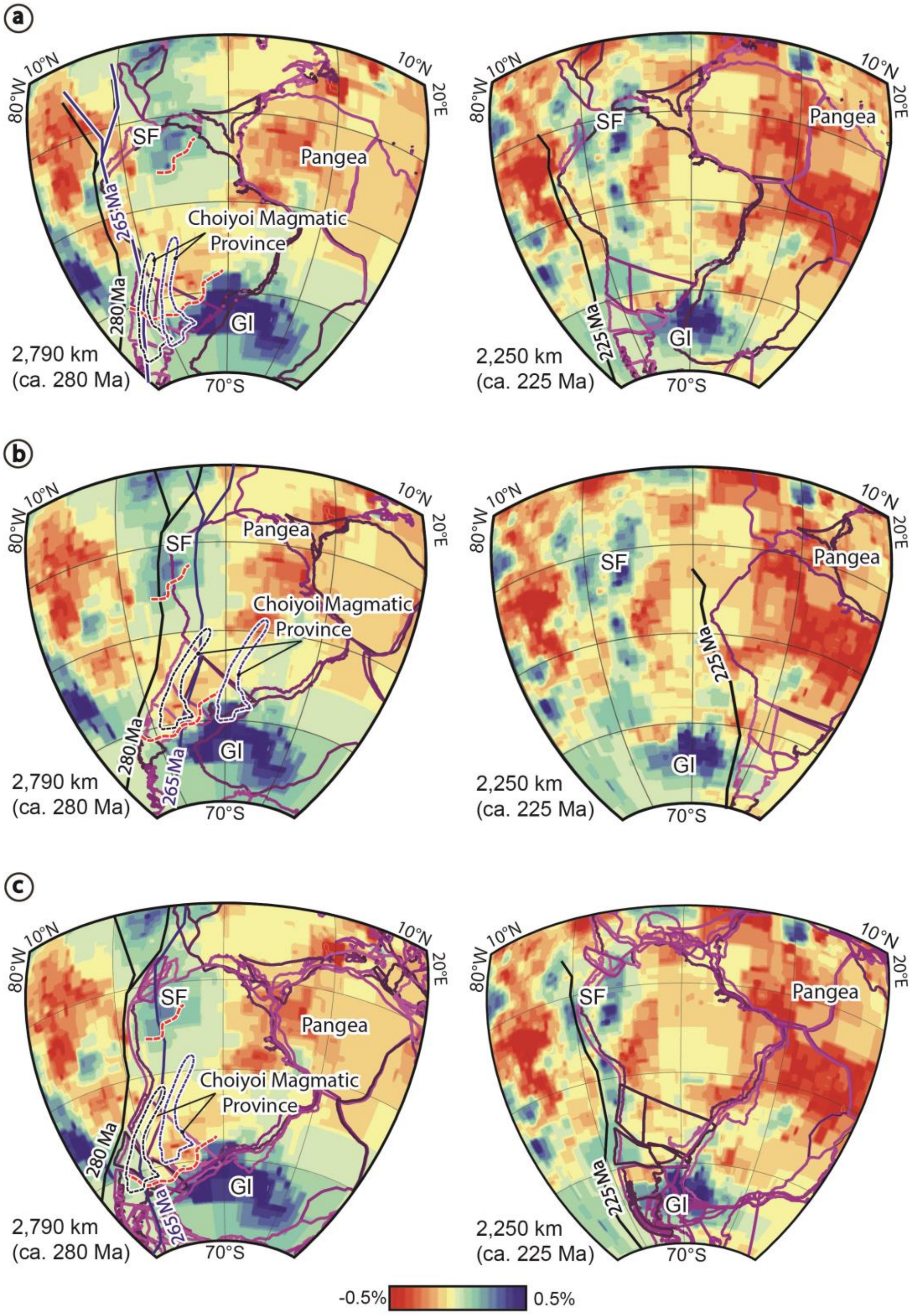

Fig. S1. Tomotectonic mantle analysis of the southwestern Pangea margin implementing plate kinematic reconstructions with alternative reference frames. a Plate reconstruction implementing the lower mantle slab reference frame of van der Meer *et al.*[39]. **b** Plate reconstruction implementing the orthoversion model of Mitchell *et al.*[49]. **c**

Plate reconstruction of Young *et al.*[50] that implements the paleomagnetic reference frame of Torsvik and Van der Voo[51]. The reconstructions include mantle tomography slices of the UU-P07 seismic tomography model[45], showing the Georgia Islands and São Francisco slabs at ca. 280 Ma, and ca. 225 Ma considering a lower mantle slab sinking rate of 1 cm/yr[38,40,42,52,54,55]. Overlaid are plate reconstructions at ca. 280 Ma and ca. 225 Ma on the left and right columns, respectively. Dotted red line indicates a high-velocity discontinuity interpreted as a major slab gap along the southwestern Pangea margin. Solid thick lines labeled with ages represent reconstructed trench positions of western South America after the restoration of Cenozoic Andean shortening[46]. Abbreviations are; GI: Georgia Islands slab, and SF: São Francisco slab.

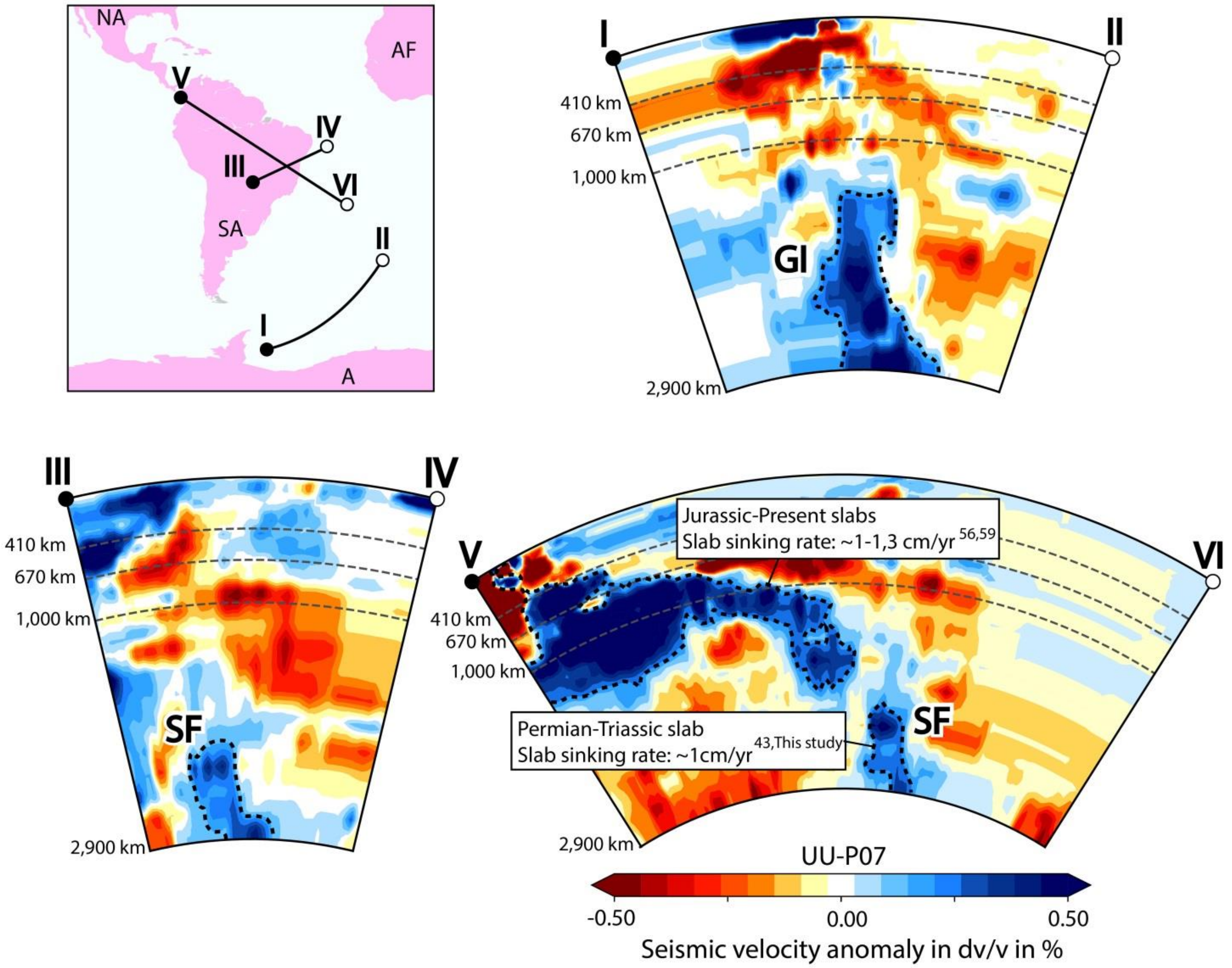

Fig. S2. Tomographic cross-sections of the UU-P07 global tomography model[45] across the São Francisco and Georgia Islands fossil slabs. Cross-sections I-II and III-IV show the wall-like geometry of the São Francisco and Georgia Islands slabs that attests for a sub-vertical slab sinking history in quasi-stationary trenches[40]. Cross-section V-VI shows the Permian to Present subducted slabs with the optimal slab sinking rates determined in previous studies. These cross-sections were built with the plotting web-tools of Hosseini *et al.*[110]. Abbreviations are; AA: South America, A: Antarctica, NA: North America, AF: Africa, SF: Sao Frãncisco Slab, and GI: Georgia Islands Slab.

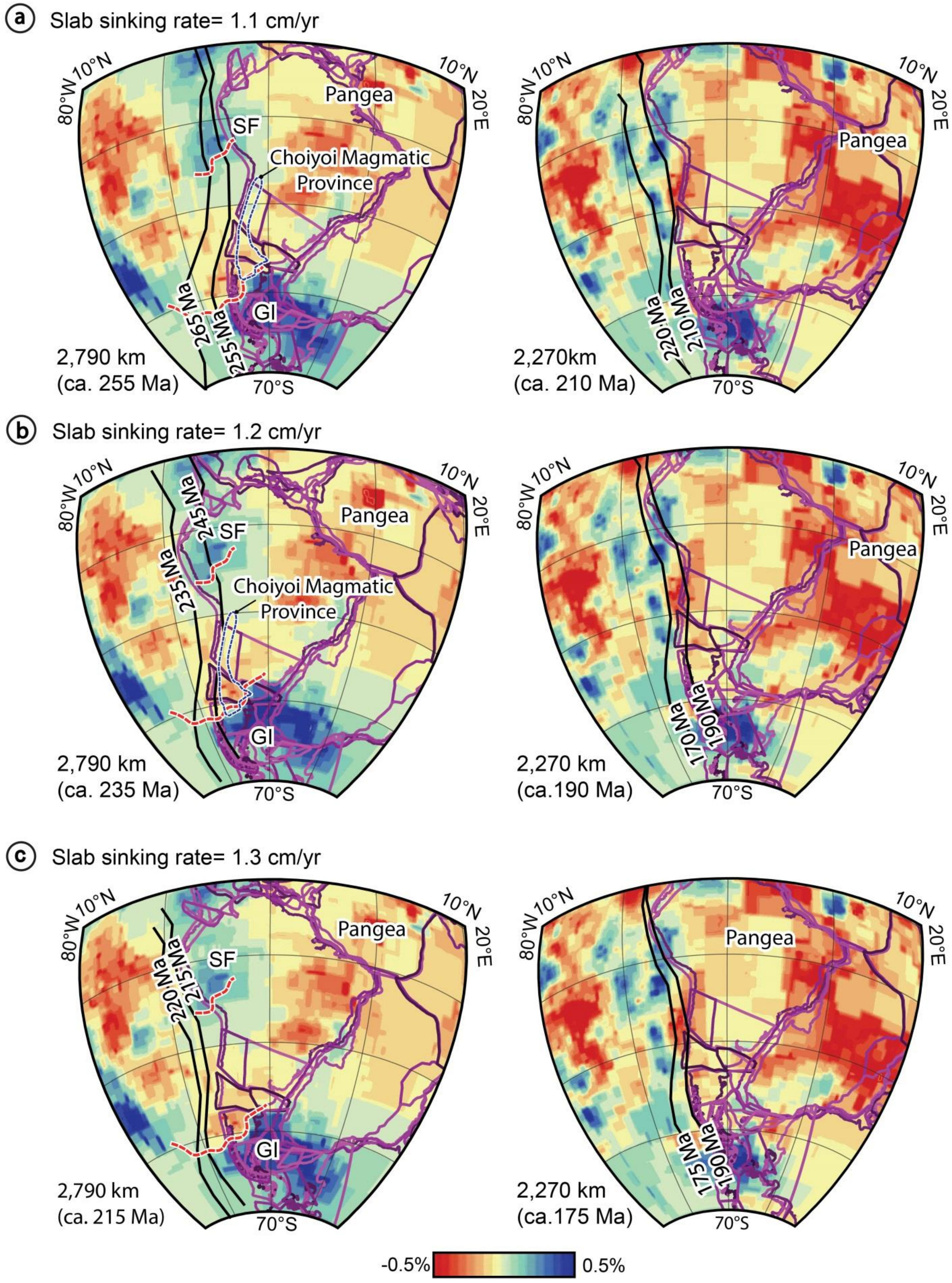

a Slab sinking rate= 1.1 cm/yr
80°W 10°N
10°N 20°E
Pangea
SF
Choiyoi Magmatic Province
265 Ma
255 Ma
GI
2,790 km (ca. 255 Ma)
70°S
220 Ma
210 Ma
2,270km (ca. 210 Ma)
b Slab sinking rate= 1.2 cm/yr
245 Ma
235 Ma
2,790 km (ca. 235 Ma)
170 Ma
190 Ma
2,270 km (ca.190 Ma)
c Slab sinking rate= 1.3 cm/yr
220 Ma
215 Ma
2,790 km (ca. 215 Ma)
175 Ma
190 Ma
2,270 km (ca.175 Ma)
-0.5%
0.5%

Fig. S3. Tomotectonic analysis taking into account additional slab sinking rates and including Paleozoic plate kinematic reconstructions from Matthews *et al.*[46]. Average slab sinking rates of **a** Shephard *et al.*[58], **b** van der Meer *et al.*[43], and **c** Butterworth *et al.*[44]. Abbreviations are; GI: Georgia Islands slab and SF: São Francisco slab.

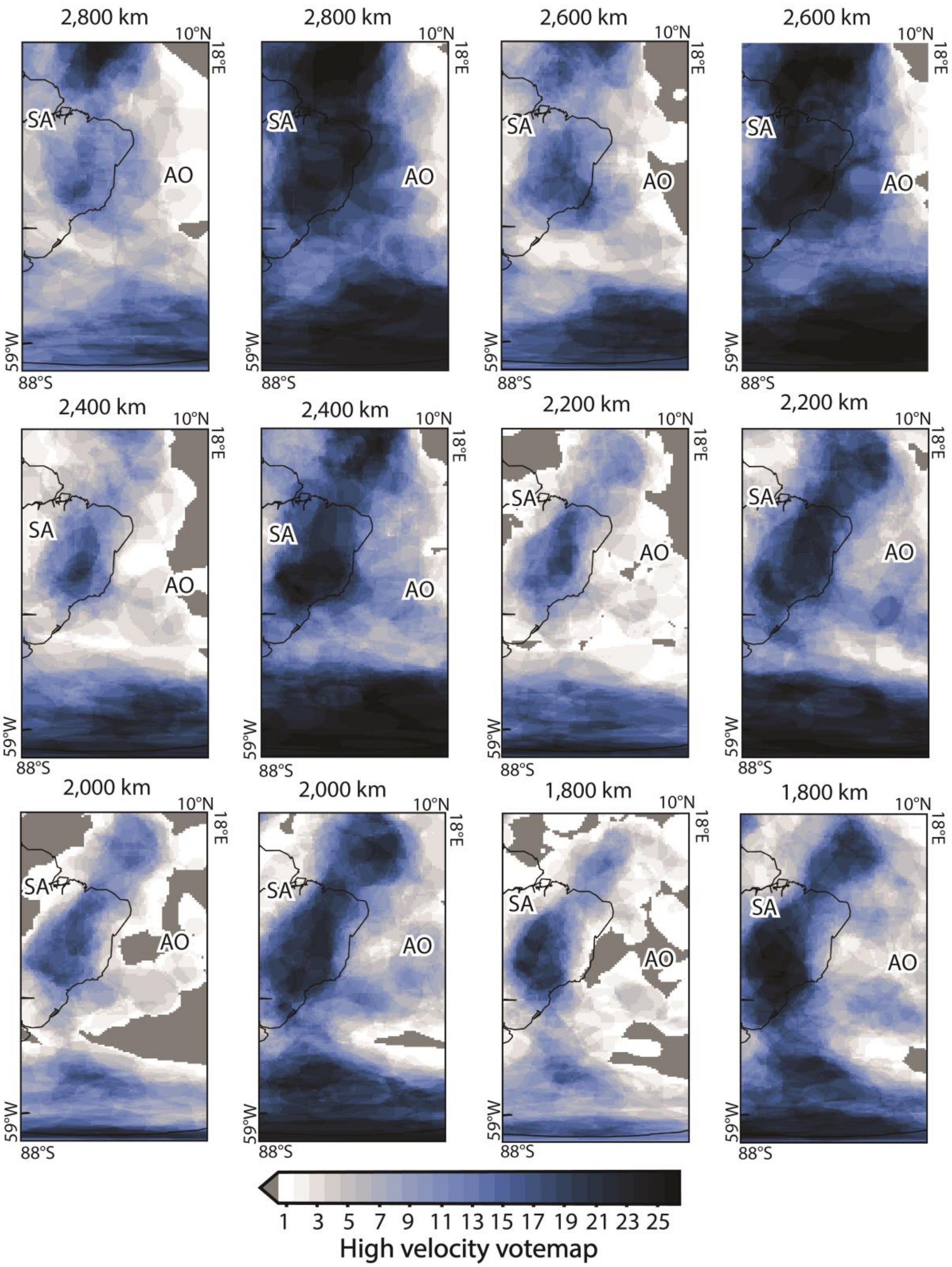

Fig. S4. High-velocity vote maps stacking 26 global seismic tomography models. For each analyzed depth, standard deviation and zero threshold metrics were implemented on the right and left maps, respectively. Abbreviations are; AO: Atlantic Ocean, and SA: South America.

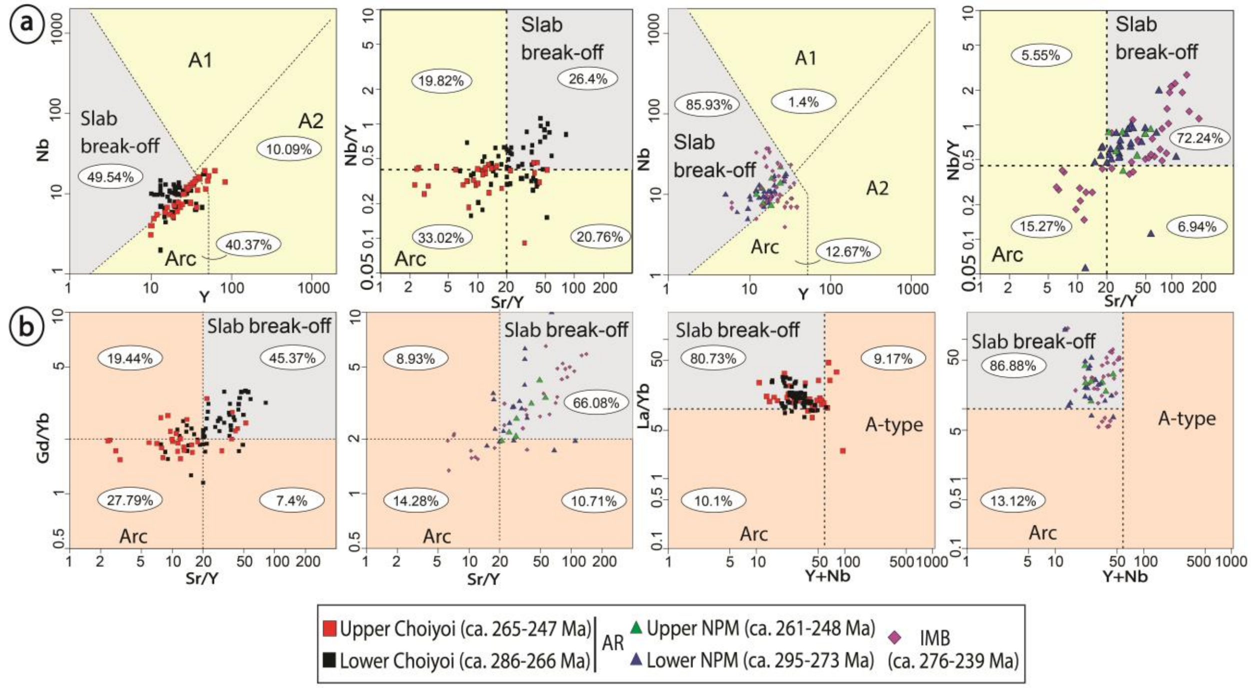

Fig. S5. **Geochemical diagrams to discriminate magmatic arc, slab break-off, and within-plate environments in mid-Permian-Lower Triassic igneous rocks along the south western Pangea margin. a** Hildebrand *et al.*[60]. **b** Whalen and Hildebrand[61]. Compiled geochemical data shows a dominant role of a slab break-off geochemical signature. Geochemical data is available in Data file S1. Abbreviations are; AR: Andean region, IMB: Intracratonic magmatic belt, and NPM: North Patagonian massif.

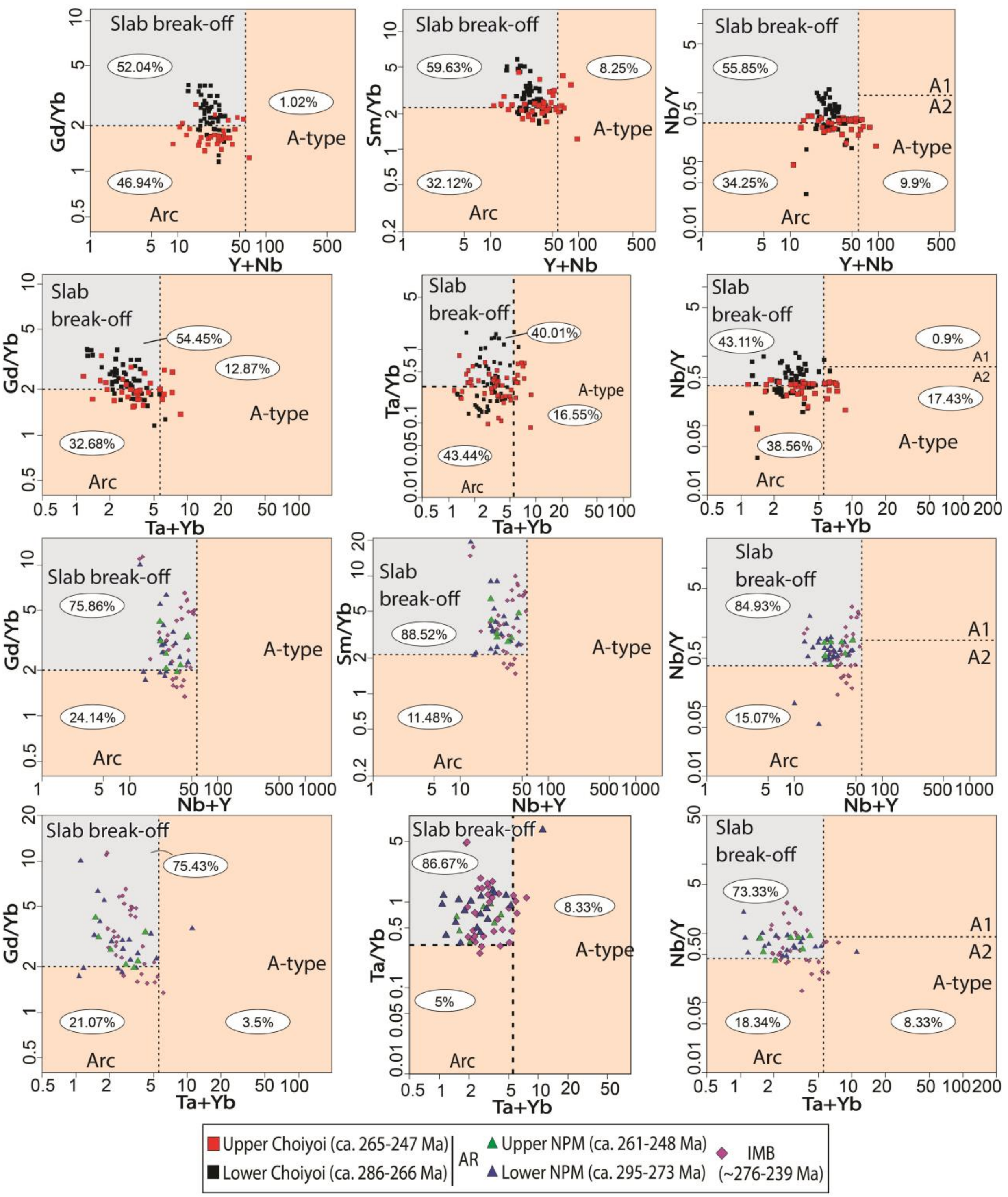

Fig. S6. Additional geochemical diagrams proposed by Whalen and Hildebrand[61] to discriminate magmatic arc, slab break-off, and within-plate in mid-Permian-Lower Triassic igneous rocks along the south western Pangea margin. The compiled geochemical data shows a dominant role of a slab break-off geochemical signature.

Geochemical data is available in Data file S1. Abbreviations are; AR: Andean region, IMB: Intracratonic magmatic belt, and NPM: North Patagonian massif.

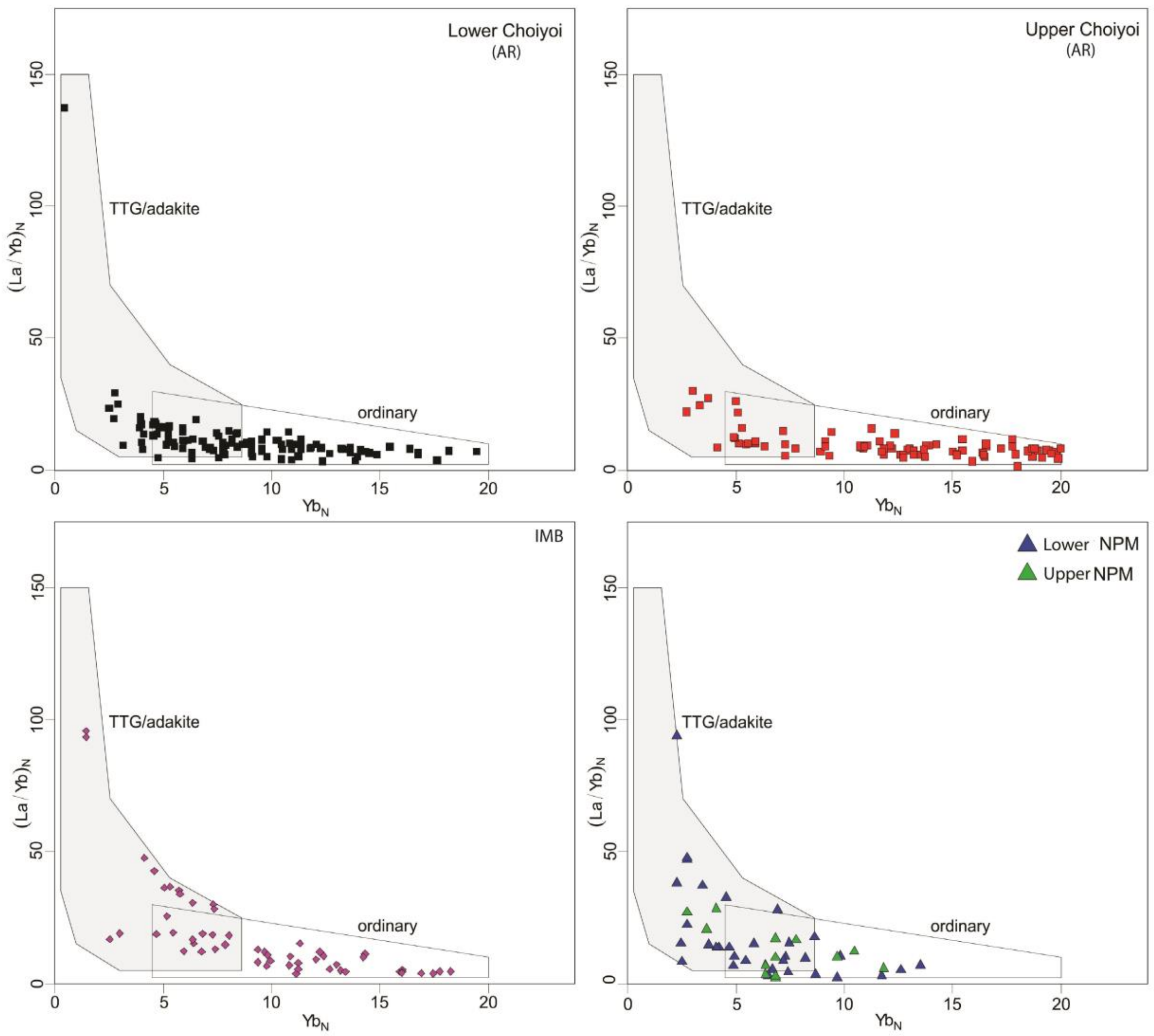

Fig. S7. (La/Yb)$_N$ vs. Yb$_N$ diagrams[67] for samples of the ChMP. This figure shows the adakitic nature of a part of the Permian-Triassic igneous rocks of the Choiyoi Magmatic Province. Geochemical data is available in Data file S1. Abbreviations are; AR: Andean region, IMB: Intracratonic magmatic belt, and NPM: North Patagonian massif.

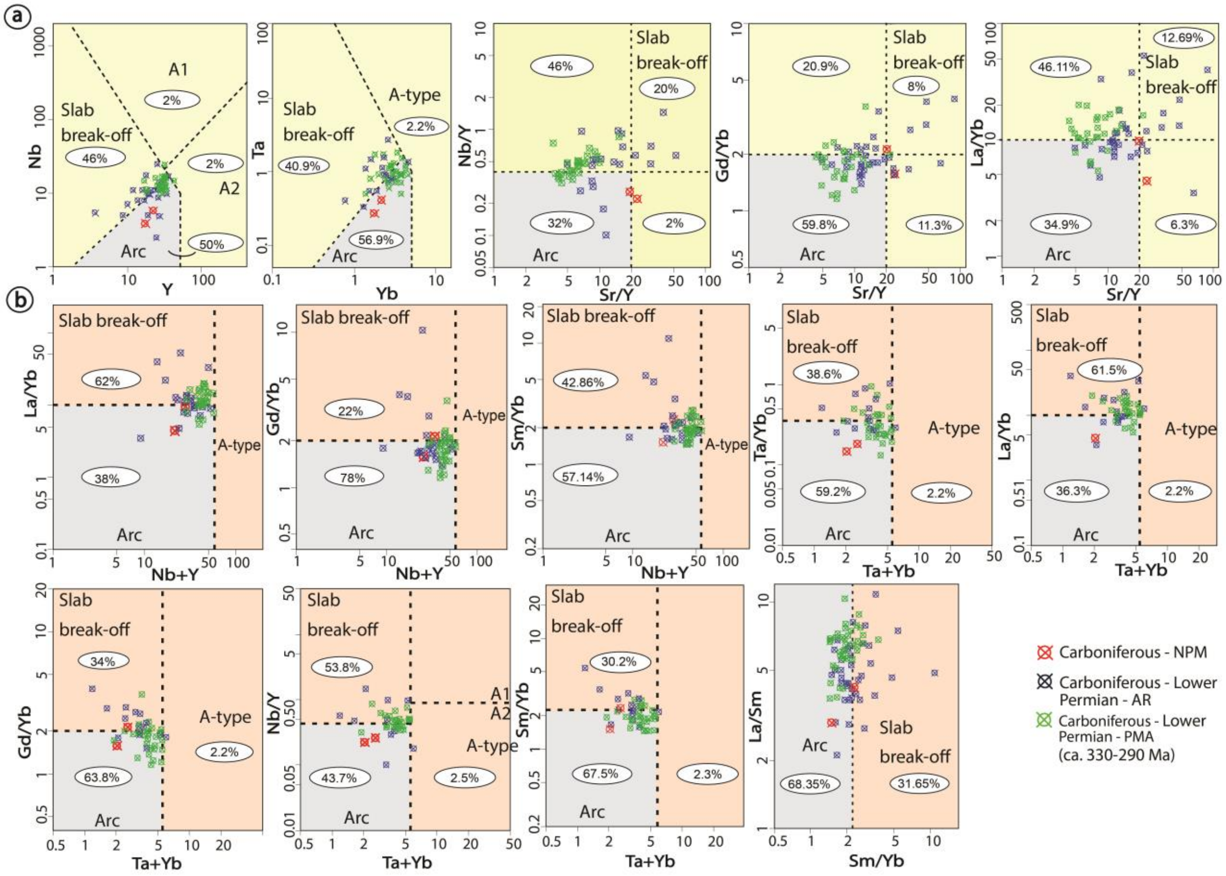

Fig. S8. **Geochemical diagrams to discriminate magmatic arc, slab break-off, and within-plate environments in Upper Carboniferous-Lower Permian igneous rocks along the south western Pangea margin. a** Hildebrand *et al.*[60]. **b** Whalen and Hildebrand[61]. Compiled geochemical data shows a dominant role of an arc geochemical signature in pre-Choiyoi igneous rocks. Geochemical data is available in Data file S1. Abbreviations are; AR: Andean region, NPM: North Patagonian massif, and PMA: Peruvian magmatic belt.

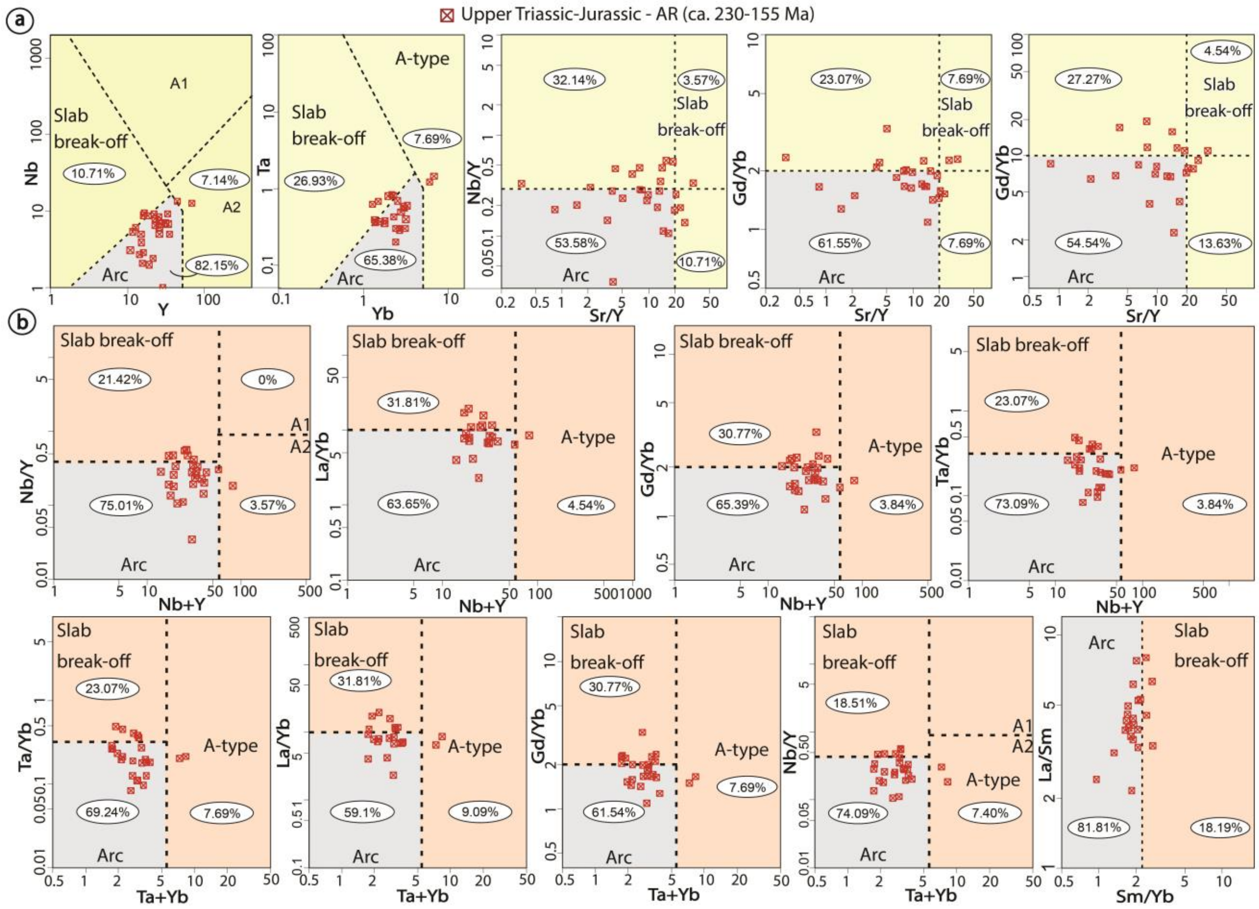

Fig. S9. **Geochemical diagrams to discriminate magmatic arc, slab break-off, and within-plate environments in Upper Triassic-Jurassic igneous rocks along the Andean region of the south western Pangea margin. a** Hildebrand *et al.*[60]. **b** Whalen and Hildebrand[61]. Compiled geochemical data shows a dominant role of an arc geochemical signature in post-Choiyoi igneous rocks. Geochemical data is available in Data file S1. AR: Andean region.

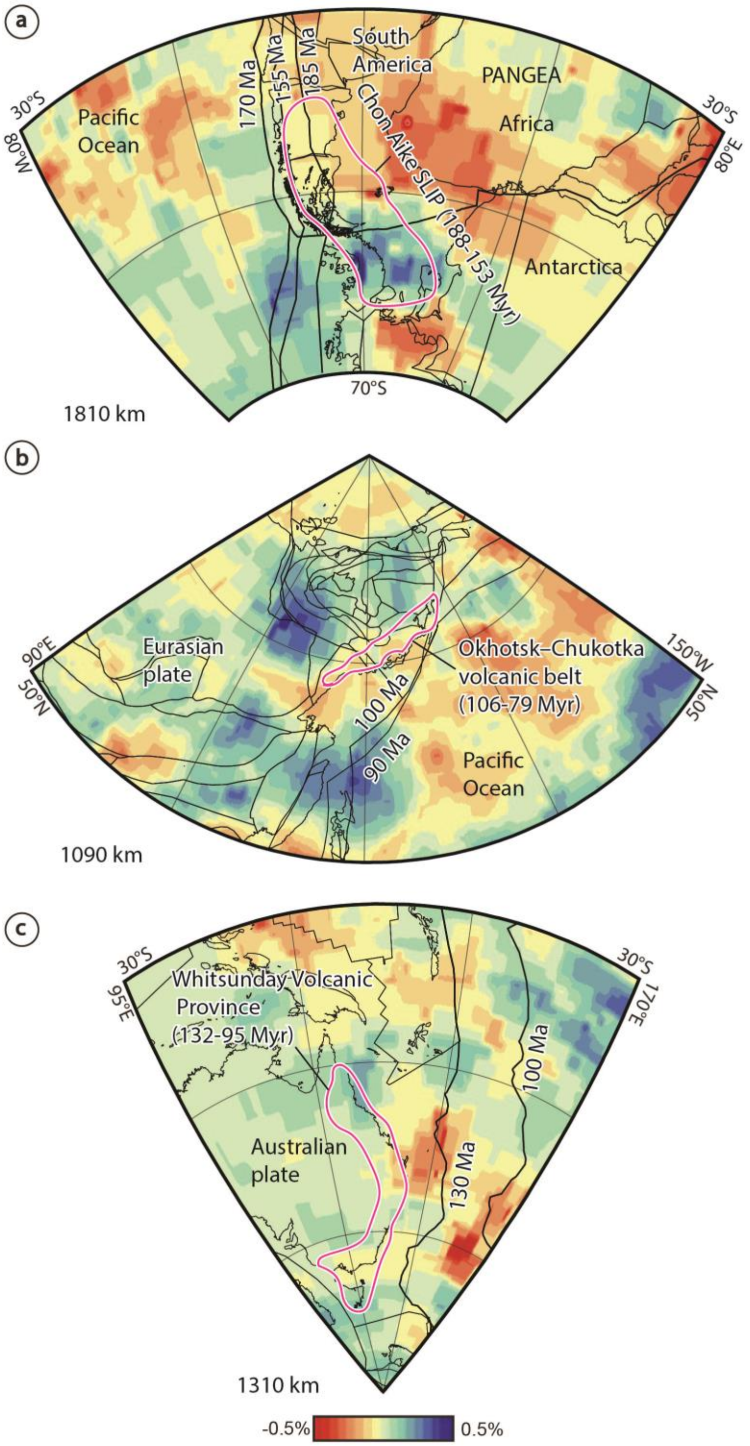

Fig. S10. Tomotectonic analysis of three different pre-Cenozoic SLIPs. a Jurassic Chon Aike magmatic province[104], **b** Upper Cretaceous Okhotsk-Chukotka volcanic belt[102], and **c** Lower to Upper Cretaceous Whitsunday volcanic province[100]. Mantle tomography slices are from the UU-P07 seismic tomography model[45] and overlaid plate reconstructions are from Mathews *et al.*[46].

Table S1. Global P- and S-wave tomography models used to construct the tomographic vote maps shown in Figs. 2d and Supplementary Fig. S1.

Model	Data type	Reference model	Model	Data type	Reference model
GyPSuM-S[111]	Body waves	TNA/SNA	MITP08[120]	Body waves	AK135
DETOX-P2[112]	Body waves (P and Pdiff)	IASP91	UU-P07[45]	Body waves	AK135
DETOX-P3[112]	Body waves (P, PP and Pdiff)	IASP91	TX2019Slab-P[121]	Body waves	AK135
HMSL-P06[113]	Surface waves, body waves	AK135 for ray tracing, traveltime measurements for each phase have the mean removed	TX2019Slab-S[121]	Body waves	TNA/SNA
HMSL-S06[113]	Surface waves, body waves	AK135 for ray tracing, traveltime measurements for each phase have the mean removed	S362ANI+M[122]	Surface waves, body waves, normal modes	STW105
PRI-P05[114]	Body waves	IASP91	S20RTS[123]	Surface waves, body waves, normal modes	PREM
PRI-S05[114]	Body waves	IASP91	S40RTS[124]	Surface waves, body waves, normal modes	PREM
SPani-P[115]	Surface waves, body waves	PREM	SAVANI[125]	Surface waves, body waves	PREM
SPani-S[115]	Surface waves, body waves	PREM	SAW642ANb[126]	Waveform	PREM
GAP-P4[116]	Body waves	GAP	SEMUCB-WM[127]	Waveform	Custom averaged model
LLNL_G3Dv3[117]	Body waves	Custom averaged model	SEMum[128]	Waveform	PREM
Hosseini2016[118]	Body waves	IASP91	TX2011[129]	Body waves	TX2011_ref
SEISGLOB1[119]	Surface waves, normal modes	PREM	TX2015[130]	Body waves	TX2011_ref
