# Supplementary material for: Catastrophic slab loss in southwestern Pangea preserved in the mantle and igneous record
Source: Nat Commun. 2022 Feb 4;13:698. doi: 10.1038/s41467-022-28290-z (PMC8817029; doi:10.1038/s41467-022-28290-z)
Supplement: Supplementary file 3 — Description of Additional Supplementary Files [file 41467_2022_28290_MOESM3_ESM.pdf]

Description of Additional Supplementary Files

File name: Supplementary Data 1

Description: Compiled geochemical data[135-156] used in Figs. 3, Supplementary Fig. S5, S6, S7, S8, and S9 (separate file)

File name: Supplementary Data 2

Description: Compiled geochronological data[5,22,62,63] used in the spatiotemporal analysis in Fig. 4 (separate file).
